# Clinical outcomes of liposomal irinotecan in advanced pancreatic adenocarcinoma patients previously treated with conventional irinotecan-based chemotherapy: a real-world study

**DOI:** 10.3389/fonc.2023.1250136

**Published:** 2023-08-28

**Authors:** Amol Gupta, Ana De Jesus-Acosta, Lei Zheng, Valerie Lee, Ihab Kamel, Dung Le, Michael Pishvaian, Daniel Laheru

**Affiliations:** The Sidney Kimmel Comprehensive Cancer Center, The Johns Hopkins Hospital, Baltimore, MD, United States

**Keywords:** pancreatic adenocarcinoma, liposomal irinotecan, irinotecan, 5-fluorouracil (5-FU) and leucovorin (LV), progression

## Abstract

**Background:**

The efficacy of combination chemotherapy beyond the first-line setting remains modest in patients with advanced pancreatic adenocarcinoma (PAC). Evidence from recent clinical studies has shown that liposomal irinotecan (nal-IRI) plus 5-fluorouracil (5-FU) and leucovorin (LV) resulted in survival benefits in patients with advanced pancreatic adenocarcinoma (APAC) after progression on gemcitabine-based treatment. However, the survival benefits of nal-IRI in the third and later lines, in which limited options are available, have yet to be extensively studied. Also, some studies have shown conflicting results regarding the impact of prior treatment with conventional IRI on patient outcomes following treatment with nal-IRI. Therefore, this real-world study aimed to evaluate the efficacy and safety of nal-IRI plus 5FU-LV in advanced PAC patients who progressed on conventional IRI-containing regimens.

**Methods:**

A retrospective chart review was conducted between November 2016 to December 2022 on 30 patients diagnosed with advanced PAC who completed at least one cycle of nal-IRI plus 5-FU- LV and were previously treated with conventional IRI. Data regarding survival outcomes were retrieved.

**Results:**

Thirty patients met the inclusion criteria. Overall, 76.7% of the patients received at least two lines of therapy prior to nal-IRI. The median overall duration of nal-IRI treatment was 2.0 months (IQR: 1.3 – 3.9 months). One patient (3.3%) had a partial response, and seven patients (23.3%) had stable disease as their best response. The median progression-free survival (PFS) was 1.9 months (95% CI 1.6 - 2.0) and the 6-month PFS rate was 20.0%. The median overall survival (OS) was 5.0 months (95% CI 3.4 – 7.0), and the 6-month OS rate was 36.7%. An interval between conventional IRI and nal-IRI ≥5.5 months was significantly associated with prolonged OS of 10.2 months (95% CI 3.3 – 12.1) versus 4.3 months (95% CI 2.1 – 5.9; p =0.003). Ten patients (33.3%) experienced grade 3 adverse events, most commonly nausea, fatigue, diarrhea, and non-neutropenic fever.

**Conclusion:**

Nal-IRI plus 5FU/LV had modest survival benefits and an acceptable safety profile in patients with prior conventional IRI. A longer interval between conventional IRI and nal-IRI was associated with increased survival outcomes.

## 1 Introduction

According to the latest estimates from the American Cancer Society (ACS), pancreatic cancer accounts for about 3% of all cancers and is the fourth leading cause of cancer related mortality in the US (1). Advanced pancreatic adenocarcinoma (PAC) is a highly aggressive malignancy with a poor prognosis due to early lymphovascular invasion and metastasis, resistance to standard chemotherapy, and asymptomatic early-stage disease (2, 3). Chemotherapy is the mainstay for treating patients with advanced PAC. The most widely used first-line chemotherapy regimens for advanced PAC are FOLFIRINOX regimen consisting of leucovorin (LV), 5-fluorouracil (5-FU), irinotecan (IRI), and oxaliplatin or gemcitabine-based (i.e. gemcitabine/nab-paclitaxel) (4–7). Randomized clinical trials have shown improved overall survival (OS) with the FOLFIRINOX regimen (median OS, 11.1 months vs 6.8 months in gemcitabine monotherapy; hazard ratio [HR] 0.57, 95% Confidence Interval [CI] 0.45 – 0.73; p <0.001) (8) and gemcitabine-based combination regimen (median OS, 8.7 months vs 6.6 months in gemcitabine monotherapy; HR 0.72, 95% CI 0.62 – 0.83; p <0.001) (9). The options are more limited in patients who progress on first-line regimens (10).

Liposomal irinotecan (nal-IRI) is a liposomal formulation of the conventional irinotecan (IRI) that was developed to improve the pharmacodynamic properties and enhance tumor exposure of conventional IRI at a lower dose (10–13). Experimental evidence demonstrated that nal-IRI led to similar tumor exposure to SN-38, the active metabolite of IRI, compared with conventional IRI at a significantly lower dose, which is assumed to be a result of the drug’s improved permeability and retention properties (14, 15). *In-vitro* and *in-vivo* studies also demonstrate greater antitumor efficacy compared to conventional IRI due to prolonged SN-38 exposure and lower drug-related systemic toxicity (13). In the phase 3 NAPOLI-1 trial, which recruited patients with metastatic PAC who progressed on gemcitabine-based treatment, nal-IRI plus 5-FU-LV led to a median OS of 6.1 months (95% CI 4.8–8.9) compared to 4.2 months (95% CI 3.3–5.3) in 5FU-LV group (HR 0.67, 95% CI 0.49–0.92; p=0·012). The median progression-free survival (PFS) was 3.1 months (95% CI 2.7 – 4.2) in patients receiving nal-IRI plus 5-FU-LV compared to 1.5 months (95% CI 1.4–1.8) in patients receiving 5FU-LV (HR 0.56, 95% CI 0.41–0.75; p=0.000) (16). The US Food and Drug Administration (FDA) approved nal-IRI in 2015 and clinical guidelines recommend nal-IRI-containing combinations as a second-line option for patients who have progressed on gemcitabine-based treatment (17, 18). Recently, the results of NAPOLI-3 trial has now shown a benefit of nal-IRI in the first-line setting in the combination 5FU/LV and oxaliplatin (NALIRIFOX) versus gemcitabine-nab-paclitaxel in both OS (11.1 vs 9.2 months; HR 0.84 [95% CI 0.71–0.99]; p = 0.04) and PFS (7.4 vs 5.6 months; HR 0.70 [0.59–0.84]; p = 0.0001) (19).

However, many questions remain unanswered despite the results of NAPOLI-1 trials. In the NAPOLI-1 trial, only 10% of the patients were previously exposed to conventional irinotecan; recent real-world evidence shows conflicting results regarding the impact of prior exposure to conventional IRI on the outcomes of nal-IRI combination treatments (20–22). Also, most of the patients treated with nal-IRI plus 5FU/LV (66%) in the NAPOLI-1 trial were in first-line or second-line in metastatic settings (16). The outcomes of nal-IRI beyond the second-line setting are unclear, even though the use of nal-IRI in this setting is increasing due to the modest efficacy of alternative options. Importantly, conflicting results are observed in real-world studies assessing the efficacy of nal-IRI in patients who received IRI. Therefore, this retrospective study involved a patient chart review investigating the effectiveness and survival outcomes of nal-IRI plus 5FU-LV in patients with advanced PAC previously treated with conventional IRI-based chemotherapy.

## 2 Materials and methods

The present study was approved by the Institutional Review Board of The Johns Hopkins Hospital (Ref No. CIR00084935). The Committee waived the need for signed written informed consent due to the retrospective nature of the study.

### 2.1 Data source and patients studied

A retrospective chart review was conducted to retrieve the de-identified patient data from the electronic medical records of the The Johns Hopkins Hospital database. Data from patients with advanced PAC who completed at least one cycle of nal-IRI plus 5-FU- LV from November 2016 to December 2022 were retrieved. The diagnosis of advanced PAC was based on pathological examination and/or imaging assessment. We limited our inclusion criteria to adults (> 18 years old) who completed at least one cycle of conventional IRI-based chemotherapy before initiating nal-IRI. Patients were excluded if they received conventional IRI in neoadjuvant or adjuvant settings, or missing data regarding post-nal-IRI computed tomography (CT) findings to extract the best response data according to the Response Evaluation Criteria in Solid Tumors (RECIST) version 1.1 criteria.

### 2.2 Patient data collection

We retrieved the following data from the records of eligible patients: demographic characteristics, tumor characteristics at diagnosis (site, stage, presence of metastasis, and histological examination findings), surgery and/or radiotherapy before nal-IRI, characteristics of prior lines of therapy (regimen, duration, number of cycles, and reason for discontinuation), the interval between discontinuation of conventional IRI and the start of nal-IRI, weight-based and total starting and cumulative doses of nal-IRI, number of nal-IRI cycles, need for dose modifications, the reason for nal-IRI discontinuation, CT findings during nal-IRI (approximately every 4 to 6 weeks), progression, mortality, and the duration of follow-up.

The duration of follow-up was calculated from the index date of starting nal-IRI until documented nal-IRI discontinuation or patient death. At our center, the standard dosing schedule of nal-IRI plus 5-FU- LV is 80 mg/m2 irinotecan hydrochloride trihydrate salt equivalent to 70 mg/m2 irinotecan free-base over 90 min, followed by 400 mg/m2 LV over 30 min and 2400 mg/m2 5-FU over 46 h, every two weeks. However, patients could start at lower doses or undergo dose modification at the treating physician’s discretion.

### 2.3 Treatment outcomes

The primary outcome of the present study was the best response according to the RECIST version 1.1 criteria. The secondary outcomes included the disease control rate (DCR), which was defined as the rate of patients who achieved complete response (CR), partial response (PR), or stable disease (SD), PFS, OS, time to progression (TTP) on nal-IRI, and the association between survival outcomes and the interval between conventional IRI and nal-IRI and the cumulative nal-IRI dose.

### 2.4 Statistical analysis

Descriptive analysis was employed according to the type of data and the normal distribution. Patient survival analysis was performed using standard Kaplan-Meier method. The Log-rank test assessed the association between survival outcomes and the interval between conventional IRI and nal-IRI. Patients were categorized into two groups according to the median interval time. The PFS and OS were defined as the time from initiating nal-IRI to disease progression and death/date of last follow-up, respectively. The TTP was defined as the time from starting the nal-IRI or IRI-containing regimens and tumor progression based on RECIST. A subgroup analysis was conducted on patients who received ≥ 2 lines of therapy for advanced PAC before nal-IRI. Cox proportional hazard regression was employed for multivariate analysis of factors associated with OS. Models were adjusted for age, gender, lines of therapy, prior surgery or radiotherapy. All analyses were conducted using JMP version 15.2 (SAS Inc. Buckinghamshire, UK) with a two-sided significance level of 0.05.

## 3 Results

### 3.1 Patient selection

**Figure 1:**
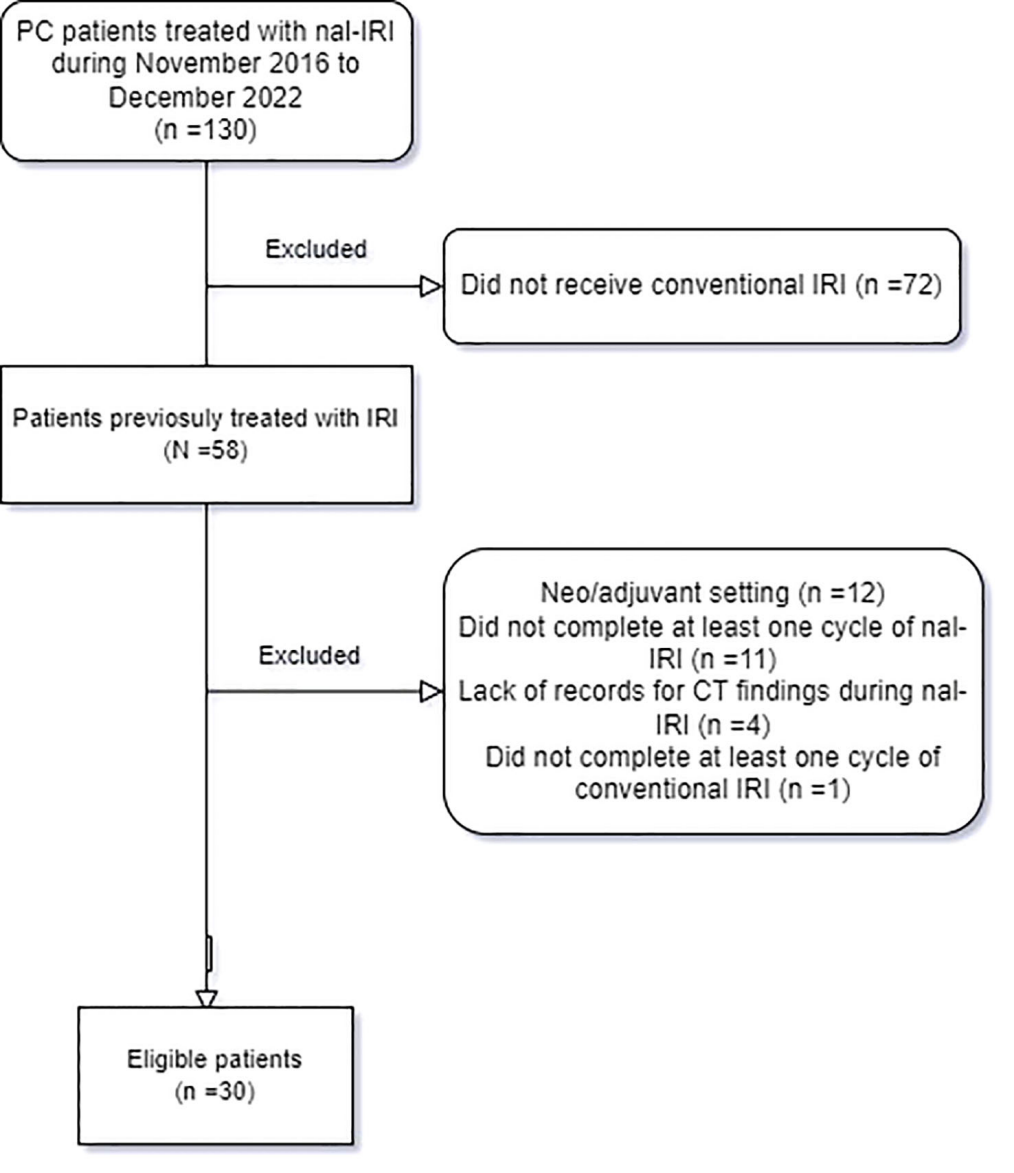
Flow diagram depicting the eligibility criteria of the study.

One hundred and thirty patients presented with advanced PAC and received nal-IRI during the data collection period. Of these, 58 patients received conventional IRI before nal-IRI. However, 12 patients were excluded as they received conventional IRI as part of neoadjuvant or adjuvant regimens only, 11 patients were excluded as they did not complete the first cycle which consists of 2 doses of nal-IRI over 28 days (they only received a single dose of nal-IRI), four patients were excluded due to missing computed tomography (CT) imaging findings during nal-IRI treatment, and one patient was excluded who did not receive at least one cycle of conventional IRI. Therefore, thirty patients were included in the retrospective chart review (**Figure 1**).

### 3.2 Characteristics of the study cohort

The median age of the included patients was 62.5 years (interquartile range [IQR] 58.8 – 71 years), and 40.0% were male patients. The primary site of PAC was the head of the pancreas (56.7%). Approximately, two-third of patients with PAC presented with metastatic disease at diagnosis. Four patients (13.3%) underwent prior surgery, and 23.3% had prior radiotherapy. Overall, 76.7% of the patients received at least two lines of therapy prior to nal-IRI. All patients received conventional IRI before initiating nal-IRI primarily as a component of FOLFIRINOX or GAX-CI (gemcitabine, nab-paclitaxel, capecitabine, cisplatin, and irinotecan) chemotherapy regimens. The most common first-line chemotherapy regimens were FOLFIRINOX/mFOLFIRINOX (66.7%). **Supplementary Table 1** shows the clinical characteristics of the patients before receiving nal-IRI.

### 3.3 Outcomes of patients treated with nal-IRI plus 5FU-LV

**Table 1** shows the characteristics and efficacy of nal-IRI plus 5FU-LV. The median interval between the discontinuation of conventional IRI and starting nal-IRI was 5.5 months (IQR: 2.7 – 8.7 months). The median starting and cumulative doses of nal-IRI were 129 mg (IQR: 102.4 – 129 mg) and 430 mg (IQR: 286 – 8114.2 mg) respectively, while the median number of completed cycles was 4 cycles (IQR: 3 – 8 cycles). Nine patients (30.0%) required dose reduction. The median overall duration of nal-IRI treatment was 2.0 months (IQR: 1.3 – 3.9 months). Twenty-six patients (86.7%) stopped the treatment due to progressive disease (PD). One patient (3.3%) was on-active treatment during data collection (**Table 1**).

**Table 1:** Treatment characteristics and clinical efficacy of liposomal irinotecan (nal-IRI) plus 5-fluorouracil (5-FU), and leucovorin (LV) regimen (N = 30 patients).

Variables		Patients (n=30)
**Interval between IRI and nal-IRI, mons**	Median (IQR)	5.5 (2.7 – 8.7)
**Duration of nal-IRI, mons**	Median (IQR)	2.0 (1.3 – 3.9)
**No. of nal-IRI cycles**	Median (IQR)	4 (3 – 8)
**Starting dose of nal-IRI (mg)**	Median (IQR)	129 (102.4 - 129)
**Cumulative dose of nal-IRI (mg)**	Median (IQR)	430 (286 – 814.2)
**Dose reduction, n (%)**		8 (26.7)
Reason for discontinuation, n (%)		
	Progression	26 (86.7)
	Adverse events	3 (10.0)
	Ongoing	1 (3.3)
Best response, n (%)		
	PR	1 (3.3)
	SD	7 (23.3)
	PD	22 (73.3)
**Median PFS (95% CI), mos**	Median (IQR)	1.9 (1.6 – 2.0)
**6-months PFS, n (%)**		6 (20.0)
**Median OS (95% CI), mos**	Median (IQR)	5.0 (3.4 – 7.0)
**6-months OS, n (%)**		11 (36.7)
**TTP on nal-IRI, mos**	Median (IQR)	1.9 (1.6 – 2.0)
**TTP with conventional irinotecan-containing chemotherapy**	Median (IQR)	6 (4.2 – 14.1)

IQR, interquartile range; TTP, time to progression; SD, stable disease; PD, progressive disease; nal-IRI, liposomal irinotecan; PFS, progression-free survival; OS, overall survival; mons, months.

One patient (3.3%) had PR, and seven patients (23.3%) had SD as their best response, according to the RECIST version 1.1 criteria. The median TTP on the nal-IRI regimen was 1.9 months (IQR: 1.6 – 2.0 months), **Table 1**.

**Figure 2:**
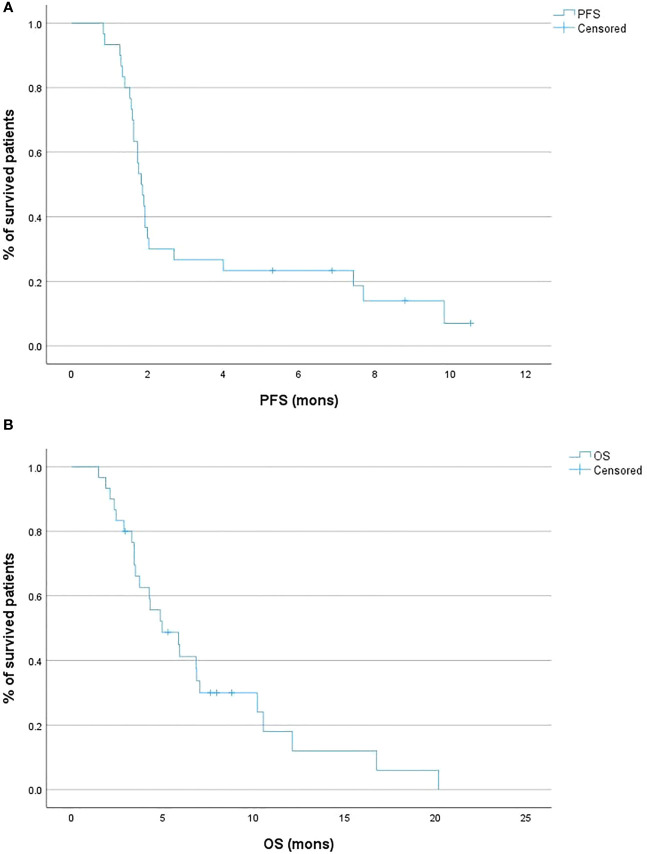
Kaplan-Meier curves of the progression-free survival (PFS) **(A)** and overall survival (OS) **(B)** of the patient cohort.

Regarding survival outcomes, the median PFS was 1.9 months (95% CI 1.6 – 2.0; **Figure 2A**), while the 6-month PFS rate was 20.0%. The median OS was 5.0 months (95% CI 3.4 – 7.0; **Figure 2B**), and the 6-month OS rate was 36.7% (**Table 1**).

**Figure 3:**
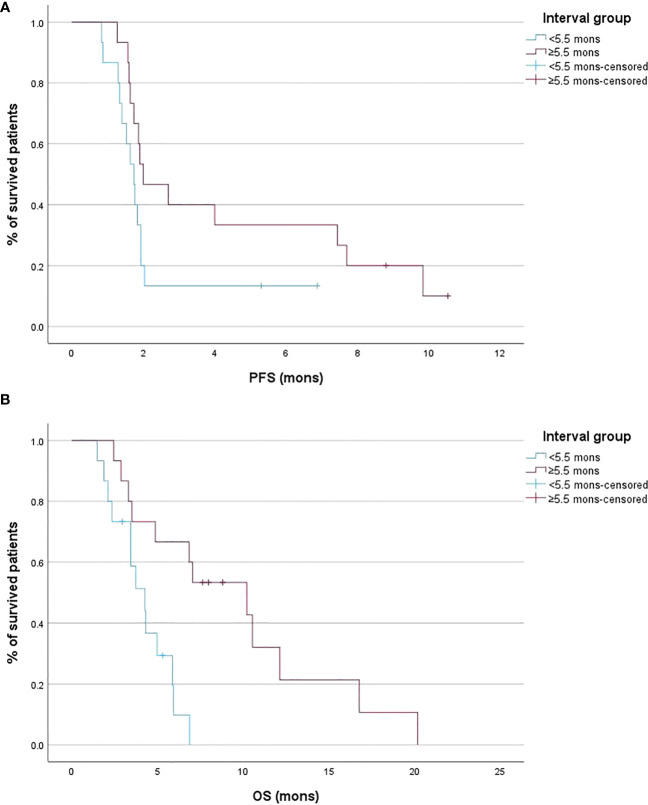
Kaplan-Meier curves of the progression-free survival (PFS) **(A)** and overall survival (OS) **(B)** stratified according to the interval between conventional irinotecan (IRI) discontinuation and liposomal irinotecan (nal-IRI) initiation.

Patients with an interval between conventional IRI and nal-IRI ≥5.5 months was had comparable PFS to patients with interval <5.5 months (2.0 months [95% CI 1.6 – 7.4] vs 1.7 months [95% CI 1.3 – 1.9], p =0.088) (**Figure 3A**). An interval between conventional IRI and nal-IRI >=5.5 months was significantly associated with prolonged OS compared to an interval of less than 5.5 months (10.2 months [95% CI 3.3 – 12.1] vs 4.3 months [95% CI 2.1 – 5.8], p =0.003; **Figure 3B**).

Univariate Cox regression analysis showed that an interval between conventional IRI and nal-IRI ≥5.5 months (HR 0.20; 95% CI 0.05 - 0.68, p =0.009) was associated with increased OS, which remained significant after multivariate analysis (p =0.024). None of the other variables, including age, gender, lines of therapy, prior surgery or radiotherapy, were significantly associated with increased OS (**Table 2**).

**Table 2:** Univariate and multivariate Cox regression of the predictors of progression-free survival (PFS) and overall survival (OS) (N = 30 patients).

Variables	PFS			OS			
	HR (95% CI)	*P-value*	aHR (95% CI)	HR (95% CI)	*P-value*	aHR (95% CI)	*P-value*
Age	0.96 (0.93 - 1.0)	0.06	–	0.98 (0.94 - 1.03)	0.25	–	–
Male gender	1.38 (0.62 - 3.08)	0.43	–	2.0 (0.59 - 5.3)	0.072	–	–
≥2 prior lines of therapy	0.99 (0.41 - 2.72)	0.98	–	2.46 (0.81 – 8.06)	0.25	–	–
Prior surgery	0.34 (0.1 - 1.0)	0.148	–	0.97 (0.25 – 3.14)	0.188	–	–
Prior radiotherapy	0.48 (0.15 – 1.2)	0.068	–	0.59 (0.16 - 1.84)	0.986	–	–
≥5.5 months between conventional and nal-IRI	0.49 (0.21 - 1.13)	0.093	–	0.20 (0.05 - 0.68)	0.009	0.20 (0.1 - 0.7)	0.024

aHR, adjusted hazard ratio; HR, hazard ratio; nal-IRI, liposomal irinotecan; IRI, irinotecan.

### 3.4 Outcomes of nal-IRI plus 5FU-LV in patients with ≥2 prior lines of therapy

**Figure 4:**
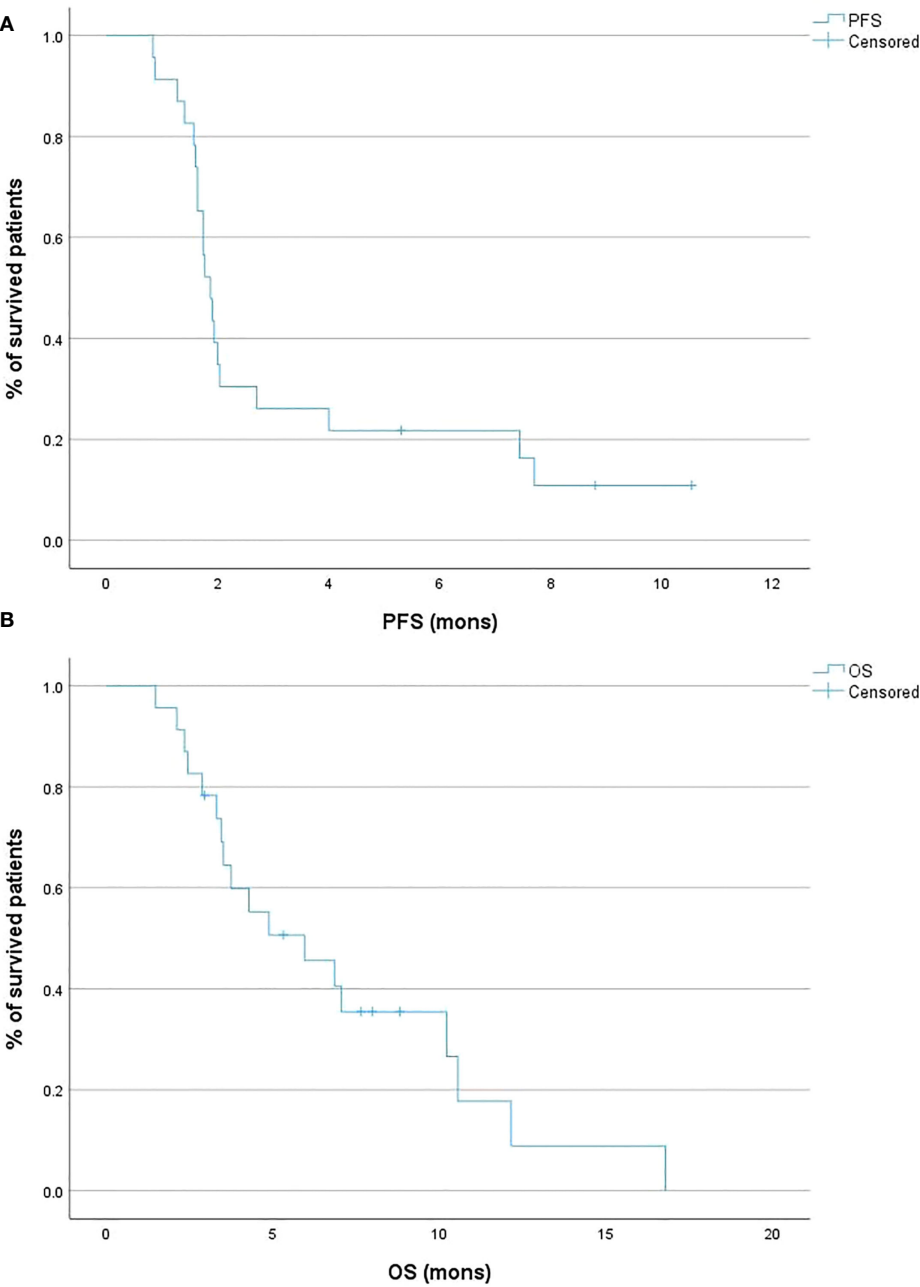
Kaplan-Meier curves of the progression-free survival (PFS) **(A)** and overall survival (OS) **(B)** according to the line of treatment of conventional irinotecan (IRI).

According to the number of prior lines of therapy, patients were divided into two groups, those with <2 (n=7) and ≥ 2 (n=23) lines of therapy. Due to the small number of patients who received <2 prior lines of therapy, we conducted survival analysis in 23 patients who received ≥ 2 lines of therapy only. One patient (4.3%) had PR, and five patients (21.7%) had SD as their best response according to the RECIST version 1.1 criteria. The median PFS was 1.9 months (95% CI 1.6 – 2.0; **Figure 4A**), and the median OS was 5.9 months (95% CI 3.4 – 10.2; **Figure 4B**).

### 3.5 Safety of nal-IRI plus 5-FU-LV

A total of 57 adverse events were observed in 66.7% (20/30) patients. Ten patients experienced a total of eleven grade 3 adverse events (AEs). The most common grade 3 AEs were diarrhea, fatigue, nausea, and non-neutropenic fever (**Supplementary Table 2**).

## 4 Discussion

In 2019, data from the NAPOLI-1 phase 3 study demonstrated the safety and clinical benefits of Nal-IRI as a second-line treatment option in patients with advanced PAC. The combination of nal-IRI and 5-FU-LV extended the survival (median improvement in OS of 1.9 months) in advanced PAC patients who are previously treated with gemcitabine-based treatment regimens (23). However, outcomes of following treatment with nal-IRI in patients with prior exposure to IRI are still unclear. In the present study, the patients’ median PFS and OS were 1.9 and 5.0 months, respectively. These findings are consistent with other retrospective studies assessing the outcomes of nal-IRI plus 5FU-LV in patients previously treated with conventional IRI. In a retrospective review from South Korea, Bang et al. found median PFS and OS were 2 and 4.4 months, respectively (22). In another retrospective study performed at Memorial Sloan Kettering Cancer Center by Glassman et al., the median PFS and OS of patients previously treated with IRI were 2.2 and 3.9 months, respectively (20). Other reports showed a median PFS of 1.6-2 months and a median OS of 4.1 months (21, 24).

These survival outcomes appear inferior to the NAPOLI-1 trial results which showed a median PFS of 3.1 months and a median OS of 6.1 months and to the findings from other studies that included patients with no prior exposure to conventional IRI (23, 25, 26). However, in the present study and similar retrospective studies, nal-IRI was administered mainly as the third-line or later lines of therapy. In contrast, nal-IRI was administrated mainly as second-line therapy in the NAPOLI-1 trial (23). In the third-line and later line treatment setting, the expected median OS is only three months with the current standard of care, while the median OS for patients in the third and later lines in the present was 5.0 months (27). Therefore, conventional IRI may not preclude the benefits of nal-IRI, particularly in the third-line and later treatment lines. This view is supported by previous comparative studies, which found no significant difference in the median OS between patients who were and were not previously treated with conventional IRI (21, 24, 28). A subgroup analysis of the NAPOLI-1 trial also found that prior exposure to conventional IRI was not an independent predictor of nal-IRI outcomes (29). Further prospective trials are needed to characterize the impact of prior exposure to conventional IRI on nal-IRI treatment outcomes in patients with advanced PAC.

Previous investigators have speculated that prior treatment with conventional IRI may be associated with developing resistance mechanisms to SN-38 in patients who have progressed, which might explain the modest efficacy of subsequent nal-IRI treatment (22). Even with the pharmacokinetics advantages of a liposomal formulation, nal-IRI-based regimens may not overcome the resistance mechanisms entirely (20, 21). A longer interval between conventional IRI and nal-IRI may allow overcoming these resistance mechanisms. Previously, it was suggested that desensitization of nuclear factor kappaB (NFκB) activation can overcome resistance to conventional chemotherapy in PAC patients (30, 31). Therefore, we hypothesized that a longer IRI-free interval may be associated with improved survival outcomes of nal-IRI treatment. The results from the present study suggested that the interval between conventional IRI and nal-IRI may influence the survival outcomes of nal-IRI. Our analysis showed that an interval between conventional IRI and nal-IRI ≥5.5 months was significantly associated with prolonged OS (10.2 versus 4.3 months). However, these findings should be interpreted cautiously as longer time between IRI exposure and subsequent nal-IRI may reflect less aggressive tumor biology, rather than an actual impact of longer interval on the response to subsequent nal-IRI. Further large-scale cohort studies are warranted to evaluate the impact of potential predictors of treatment response, including the treatment interval between conventional IRI and nal-IRI, on the outcomes of nal-IRI-based regiments in patients with advanced PAC.

The safety profile of nal-IRI has also been a point of consideration. In the NAPOLI-1 trial, the most common grade 3 or higher AEs observed in the nal-IRI plus 5FU-LV group were neutropenia, diarrhea, vomiting, and fatigue (23). Such findings align with our real-world experience, in which ten patients (33.3%) experienced grade 3 AEs, most commonly diarrhea, fatigue, nausea, and non-neutropenic fever. Similar results have been reported in other real-world studies (22). Nal-IRI has shown a favorable safety profile compared with conventional irinotecan, emphasizing its potential as a viable treatment option for advanced PAC (15). In patients with metastatic PAC in the second-line setting, nal-IRI was associated with lower incidences of pancytopenia and similar or lower uses of medications to manage AEs than FOLFIRI, FOLFIRINOX, and FOLFOX (32–35). Single-agent docetaxel (another option for advanced PAC) can be associated with significant grade 3 toxicities, such as pancytopenia, fatigue, and neuropathy (36). Therefore, the toxicities seen with a nal-IRI-based combination are relatively modest compared with other combination chemotherapy regimens.

Although the present study provided novel insights regarding the effectiveness of nal-IRI plus 5FU-LV in patients previously treated with conventional IRI, this study had several limitations. Our findings depend on retrospective data collection, which increases the risk of misclassification and recall bias. Second, the study represents a single-center experience with a relatively small sample size. The small sample size in our study might have contributed to the lower prevalence of male gender in our study compared to similar real-world experiences. This may affect the generalizability of our findings. Additionally, modifications in treatment regimens and assessment intervals were based solely on the treating physician’s discretion, which could have impacted the homogeneity of our study cohort. Patients who received conventional IRI in the neoadjuvant or adjuvant settings only were excluded as the response to therapy and subsequent clinical outcomes might be significantly different between patients who received conventional IRI in the neo/adjuvant settings and those treated in the metastatic setting. In the neo/adjuvant setting, the disease burden is generally lower, and patients are often in better overall health status at the time of IRI exposure, factors that could influence the treatment response and clinical outcomes. Additionally, data regarding CA19-9 response were not collected at appropriate time intervals to allow for statistical comparison of the response to nal-IRI. Lastly, data on the sites of metastasis, particularly distinguishing between lung-limited and other metastases, best response to prior IRI, the administration of growth factor support, and UGT1A1 genotype were not systematically available in our medical records.

## 5 Conclusion

This retrospective study from a single center of patients with advanced PAC showed that treatment with nal-IRI plus 5FU/LV resulted in modest survival benefits and an acceptable safety profile in patients with prior exposure to conventional IRI. The similar patient survival outcomes following nal-IRI treatment and the superior safety profile to alternate treatment regimens supports its use as a third-line or later treatment choice. The increased interval between conventional IRI and nal-IRI may be associated with better improved patient survival outcomes, which supports a personalized treatment plan that incorporates nal-IRI-based regimens at later stages for selected patients previously treated with conventional IRI. Further large-scale cohort studies are warranted to evaluate the impact of potential predictors on the outcomes of nal-IRI-based regiments in patients with advanced PAC.

## Data availability statement

The original contributions presented in the study are included in the article/**Supplementary Material**. Further inquiries can be directed to the corresponding authors.

## Ethics statement

The studies involving humans were approved by Institutional Review Board of The Johns Hopkins Hospital (Ref No. CIR00084935). The studies were conducted in accordance with the local legislation and institutional requirements. Written informed consent for participation was not required from the participants or the participants’ legal guardians/next of kin in accordance with the national legislation and institutional requirements.

## Author contributions

Conception and design: AG, DLa. Administrative support: all authors. Provision of study materials or patients: all authors. Collection and assembly of data: all authors. Data analysis and interpretation: all authors. Manuscript writing: AG, DLa. All authors contributed to the article and approved the submitted version.
