# Supplementary material for: Clinical outcomes of liposomal irinotecan in advanced pancreatic adenocarcinoma patients previously treated with conventional irinotecan-based chemotherapy: a real-world study
Source: Front Oncol. 2023 Aug 28;13:1250136. doi: 10.3389/fonc.2023.1250136 (PMC10494436; doi:10.3389/fonc.2023.1250136)
Supplement: Supplementary file 1 [file DataSheet_1.pdf]

1 Supplementary Tables

Table 1. Baseline characteristics of the included patients before starting liposomal irinotecan (nal-IRI) (N = 30 patients)

**Variables**		**Patients (n=30)**
**Age, y**	Median (IQR)	62.5 (58.8 – 71)
**Sex, n (%)**		
	Female	18 (60.0)
	Male	12 (40.0)
**Site, n (%)**		
	Head	17 (56.7)
	Body	6 (20.0)
	Tail	4 (13.3)
	Neck	2 (6.7)
**Stage at diagnosis, n (%)**		
	III	10 (33.3)
	IV	20 (66.7)
**Metastatic disease at nal-IRI initiation, n (%)**		27 (90.0)
**Prior surgery, n (%)**		4 (13.3)
**Prior radiotherapy, n (%)**		7 (23.3)
**Prior lines of therapy before nal-IRI, n (%)**		
	1	7 (23.3)
	2	18 (60.0)

	3	5 (16.7)
**Lines containing conventional IRI, n (%)**		
	1	26 (86.7)
	2	3 (10.0)
	3	1 (3.3)
**Reasons for discontinuation of the conventional IRI, n (%)**		
	Completion	3 (10.0)
	Intolerance	3 (10.0)
	Progression	24 (80.0)
**First-line regimen, n (%)**		
	FOLFIRINOX	8 (26.7)
	mFOLFIRINOX	12 (40)
	Gemcitabine, docetaxel and capecitabine	1 (3.3)
	Gemcitabine, nab-paclitaxel, capecitabine, cisplatin, and irinotecan	5 (16.7)
	Gemcitabine, docetaxel, capecitabine, cisplatin	1 (3.3)
	Nab-paclitaxel and gemcitabine	1 (3.3)
	Gemcitabine, nab-paclitaxel, capecitabine, cisplatin, and irinotecan, followed by maintenance of Pembrolizumab and Olaparib	1 (3.3)
	J1847 (gemcitabine/nab-paclitaxel/xeloda/cisplatin/irinotecan)	1 (3.3)

**Reasons for discontinuation of the first line, n (%)**	Completion	3 (10.0)
	Intolerance	3 (10.0)
	Progression	24 (80.0)
		**(n =23)**
**Second-line regimen, n (%)**	Cabiralizumb + Nivolumab	1 (4.3)
	Capecitabine	2 (8.7)
	Gemcitabine	1 (4.3)
	Gemcitabine, Cisplatin	2 (8.7)
	Irinotecan and oxaliplatin	1 (4.3)
	mFOLFIRINOX	2 (8.7)
	Gemcitabine, docetaxel and capecitabine	1 (4.3)
	Gemcitabine, docetaxel, capecitabine, and Cisplatin	1 (4.3)
	nab-Paclitaxel and gemcitabine	9 (39.1)
	Metformin and Rapamycin	1 (4.3)
	Nivolumab and Ipilimumab with or without GVAX Pancreas Vaccine (with Cyclophosphamide)	1 (4.3)
	Radiotherapy plus capecitabine	1 (4.3)
		**(n =23)**
**Reasons for discontinuation of the second line, n (%)**	Intolerance	2 (8.7)
	Progression	21 (91.3)
		**(n =5)**

**Third-line regimen, n (%)**	FOLFIRI	1 (20.0)
	Gemcitabine and Carboplatin	1 (20.0)
	Nab-paclitaxel and gemcitabine	3 (60.0)
		**(n =5)**
**Reasons for discontinuation of the third line, n (%)**	PD	5 (100.0)

Abbreviations: IQR: interquartile range; nal-IRI: liposomal irinotecan; IRI: irinotecan; PD: progression of disease; FOLFIRINOX: folinic acid, fluorouracil, irinotecan hydrochloride, and oxaliplatin; mFOLFIRINOX: modified folinic acid, fluorouracil, irinotecan hydrochloride, and oxaliplatin; FOLFIRI: folinic acid, fluorouracil, and irinotecan hydrochloride.

Table 2: Rate of adverse events (AEs) (N = 30 patients)

	**All grade**	**Grade ≥3**
No. of patients with AE	20 (66.7%)	10 (33.3%)
**AEs**		
Anemia	3 (10%)	0
Diarrhea	11 (36.7%)	2 (6.7%)
Dry skin	1 (3.3%)	0
Fatigue	10 (33.3%)	2 (6.7%)
Mucositis	2 (6.7%)	1 (3.3%)
Nausea	7 (23.3%)	2 (6.7%)
Neutropenia	1 (3.3%)	0
Neutropenic Fever	1 (3.3%)	1 (3.3%)
Non-neutropenic Fever	3 (10%)	2 (6.7%)

Pedal edema	1 (3.3%)	0
Peripheral neuropathy	1 (3.3%)	0
Vomiting	5 (16.7%)	1 (3.3%)
